# Interferon-alpha, immune activation and immune dysfunction in treated HIV infection

**DOI:** 10.1038/cti.2014.1

**Published:** 2014-02-28

**Authors:** Lilian Cha, Cassandra M Berry, David Nolan, Allison Castley, Sonia Fernandez, Martyn A French

**Affiliations:** 1 School of Pathology and Laboratory Medicine, University of Western Australia, Crawley, Western Australia, Australia; 2 School of Veterinary and Life Sciences, Murdoch University, Murdoch, Western Australia, Australia; 3 Department of Clinical Immunology and Pathwest Laboratory Medicine, Royal Perth Hospital, Perth, Western Australia, Australia

**Keywords:** HIV, immune activation, interferon-alpha, monocytes, type I interferons

## Abstract

Type I interferons (IFNs) exert anti-viral effects through the induction of numerous IFN-stimulated genes and an immunomodulatory effect on innate and adaptive immune responses. This is beneficial in controlling virus infections but prolonged IFN-α activity in persistent virus infections, such as HIV infection, may contribute to immune activation and have a detrimental effect on the function of monocytes and T and B lymphocytes. Activation of monocytes, associated with increased IFN-α activity, contributes to atherosclerotic vascular disease, brain disease and other ‘age-related diseases' in HIV patients treated with long-term antiretroviral therapy (ART). In HIV patients receiving ART, the anti-viral effects of IFN-α therapy have the potential to contribute to eradication of HIV infection while IFN-α inhibitor therapy is under investigation for the treatment of immune activation. The management of HIV patients receiving ART will be improved by understanding more about the opposing effects of IFN-α on HIV infection and disease and by developing methods to assess IFN-α activity in clinical practice.

Infection by HIVs and simian immunodeficiency viruses (SIVs) causes immune dysfunction in hosts that are susceptible to disease. Depletion of CD4^+^ T cells is the most important immune defect and the major contributor to AIDS that these infections cause. Direct infection and destruction of CD4^+^ T cells, particularly the central memory subpopulation, contributes to the depletion of CD4^+^ T cells, especially in early HIV infection. However, immune activation and activation-induced cell death are also major contributors to this process. Immune activation is also associated with chronic inflammation and activation of the coagulation system that increase the risk of ‘serious non-AIDS events' such as atherosclerotic vascular disease, osteoporosis, osteonecrosis and chronic kidney disease.^1^ Inflammation also affects lymphoid tissue where the resultant tissue fibrosis is a cause of impaired homoeostasis of CD4^+^ T cells^2^ and probably memory B cells.

Suppression of HIV infection by combination antiretroviral therapy (ART) decreases immune activation and inflammation and increases CD4^+^ T-cell numbers in most patients. However, CD4^+^ T-cell deficiency persists in up to 40% of patients receiving ART, particularly those patients who commenced ART with a very low CD4^+^ T-cell count,^3^ and is associated with persistent immune activation and an increased risk of ‘serious non-AIDS events'. A substantial amount of evidence implicates increased interferon-alpha (IFN-α) activity as a cause of immune activation and immune dysfunction in HIV patients. Persistence of these immune defects in patients receiving ART might therefore be amenable to therapeutic blockade of IFN-α or its receptors. However, managing this therapy in clinical practice will be problematic, because IFN-α also possesses anti-viral effects that may be beneficial in the control of HIV infection and other virus infections.

## IFN-α in antiviral responses

IFN-α consists of a family of type I IFNs that are encoded by 13 IFN-α subtype genes in humans,^4^ who also possess one IFN-β, IFN-ɛ, IFN-κ and IFN-ω gene. Human IFN-α proteins are encoded by intronless genes clustered on human chromosome 9 and share an α-helical structure of approximately 80% conserved 166 amino acids. Most IFN-α subtypes are inducible, acid stable and exhibit antiviral, antiproliferative and immunomodulatory activities.^4, 5^ Differential expression profiles of type I IFN subtypes have been found in rhesus macaques infected with SIV,^6^ but the effects of different IFN-α subtypes in the pathogenesis of SIV and HIV disease is unclear. Despite different biological effects, all type I IFNs share a surface receptor composed of two IFN-α receptor subunits (IFNAR1 and IFNAR2), which can be either anchored in the cell membrane or shed as a truncated soluble form.^7^

Normal type I IFN signalling pathways begin with ligand binding through the receptor chains to form a stable ternary complex, followed by activation of the Janus (Jak) tyrosine kinases (Tyk2 and Jak1). Subsequent attraction and phosphorylation of signal transducer and activator of transcription (STAT) molecules leads to the formation of heterodimers, which disassociate from the receptors and translocate to the nucleus to complex with interferon regulatory factor 9 (IRF9), which in turn forms the transcription factor interferon-stimulated gene factor 3 (ISGF3) that activates transcription of IFN-stimulated genes (ISGs) via recognition of upstream sequence elements in promoters. Differential biological activities of IFN subtypes may be explained in part by signalling differences in STAT and mitogen-activated protein kinase activation, with various tyrosine phosphorylation events found among the IFN subtypes and/or an association of different binding affinities of subtypes to their receptor.^8^

The overall antiviral activities of type I IFNs are complex with individual human IFN-α subtypes having been found to differ in their antiviral potency associated with virus-specific expression levels of ISG subsets, and some ISGs being antiviral while others actually enhance virus replication *in vitro*.^9^ In our studies of retroviruses in mice, the antiviral activities of subtypes *in vitro* did not always correlate with *in vivo* activities during acute Friend virus infection, with IFN-α5 and IFN-α6 incapable of reducing viral loads. Indeed, the antiviral capacity of IFN subtypes IFN-α1, IFN-α4, IFN-α9 and IFN-α11 were shown to depend on ISG expression as well as enhancement of natural killer (NK) cell and virus-specific CD8^+^ T-cell responses.^5^ It is noteworthy that most viruses have evolved strategies to evade type I IFN responses through mechanisms of direct blockade of induction, inhibition of signalling pathways or prevention of ISG expression. Perhaps numerous IFN-α subtypes have been maintained throughout evolutionary history as a survival strategy: an arsenal of responses against different viruses with specific type I IFN subtypes capable of inducing subsets of ISGs that are more effective at inhibiting different viruses.

Although viruses can evade immediate antiviral activities of type I IFNs, IFN-α has significant therapeutic potential, in combination with anti-viral drugs, for controlling infections by viruses, including HIV. However, low levels of virus persistence during chronic viral infections, especially in immune cells which are not compartmentalized, may influence the immunomodulatory effects of type I IFNs. During disease progression, modification of type I IFN pathways, either virus or immune-mediated, results in lower levels of IFNAR occupancy producing responses that may lead to immunopathology.

## Biological properties of IFN-α

Although original interest was in the antiviral properties of type I IFNs, many ISGs have also been linked to the pathogenesis of infectious disease, inflammatory disorders, autoimmune diseases and cancers.^10^ The regulation and modulation of type I IFN production is intricate and involves a cascade of sensor molecules, adaptors, kinases and transcription factors, which drive the acute innate response and sculpt the adaptive immune response. Redundancy in type I IFN signalling pathways coupled with their pleiotropic properties make definitive roles of such molecules elusive at the single cell level. The avidity of binding, competition for cytosolic molecules and timing of activation by kinase phosphorylation all influence production levels of type I IFNs.^10^

The products of many virus-induced ISGs are antiviral and clearly established biomarkers found in the blood of patients with productive virus infections. Other functions of IFN-α include modulation of both innate and adaptive immune responses, activating monocytes, antigen-presenting cells, dendritic cells (DCs), macrophages, NK cells, T cells and B cells, with stimulation of antibody class switching.^11^ Cross-talk between type I IFNs is associated with an ISG expression signature dependent on cell types. For example, exposure of macrophages to continuous low levels of IFN-α leads to enhanced IFN-γ induction of interleukin (IL)-12p70 through favoured STAT1 homodimer formation, in contrast to exposure to high levels of type I IFN, which inhibits IFN-γ-induced activation of major histocompatibility complex (MHC) class II expression and dampens IL-12p70 production.^12^ Thus, virus infections induce immune responses by eliciting production of type I IFNs, which in turn exert effects that include sustained clonal expansion, differentiation and survival of proliferating cells. However, type I IFNs may also exert pro-apoptotic and anti-proliferative effects and sustained production may adversely affect cell viability and functions such as autophagy, cell migration and vasculogenesis. Thus, type I IFN has a critical role in survival from an acute virulent virus infection, but in persistent viral infections, chronic exposure to type I IFN activity may impair ‘protective' immune responses against the virus^13^ and result in host susceptibility to collateral damage.

## Control of HIV infection by IFN-stimulated genes

Numerous intracellular anti-viral restriction factors contribute to the control of HIV infection, including several that are ISGs. These include apolipoprotein B mRNA-editing, enzyme-catalytic, polypeptide-like 3 family proteins, which restrict HIV replication in DCs,^14^ MX2, which exerts post-entry inhibition of HIV infection,^15, 16^ and bone marrow stromal cell antigen 2 (also known as tetherin), which restricts release of HIV-1 from infected cells.^17^

## IFN-α and immune dysfunction in HIV and SIV infection

Immune activation is a characteristic of HIV disease and has been most clearly demonstrated by the altered expression of ‘activation markers', such as CD38 and HLA-DR, on the surface of T cells. However, the underlying causative mechanisms remain unclear. There is strong evidence that microbial translocation from the gut, co-infections (for example, cytomegalovirus, hepatitis C virus and hepatitis B virus) and persistent low-level HIV replication contribute to residual immune activation to one degree or another in treated HIV patients.^1^ All can induce IFN-α activity and ISG expression, which may elevate other markers of immune activation and promote disease progression.

Many different cells produce IFN-α but plasmacytoid DCs (pDC) produce up to 1000-fold more than other cell types. As pDC express the CD4 receptor, they are susceptible to infection by HIV via binding of the gp120 envelope protein of the HIV virion to CD4. Consequently, frequencies of peripheral pDC decline as HIV infection progresses,^18, 19, 20^ and this loss may not be reversed with the administration of ART.^21, 22^ Loss of pDC is thought to be a result of both the apoptosis of pDC following their direct infection by HIV^23^ and the redistribution of pDC from the peripheral circulation to secondary lymphoid organs in response to HIV-induced upregulation of the migration marker CCR7.^24, 25^

Despite the reduction in circulating pDC frequencies, IFN-α production is markedly increased in acute and chronically infected HIV-positive individuals as a result of HIV-induced pDC hyperactivation. Several studies have documented an increase in IFN-α mRNA from peripheral pDCs in HIV infection, complemented by markedly higher IFN-α levels in serum from untreated acutely and chronically infected patients.^20, 26, 27^ HIV infection may even induce a distinct signature of IFN-α activity, with evidence suggesting that specific upregulation of the IFN-α subtypes, IFN-α2 and IFN-α6, is observed in both the acute and chronic phases of untreated disease.^28^

Correspondingly, ISGs are upregulated in CD4^+^ and CD8^+^ T-cell subpopulations,^29, 30, 31^ monocytes and DCs^31, 32^ in untreated HIV infection. The amount of ISGs positively correlates with HIV viral load and inversely correlates with CD4^+^ T-cell counts.^31^ Further evidence of a role for increased IFN-α activity in causing HIV-induced immune dysfunction has been obtained from animal models; persistent upregulation of ISGs in CD4^+^ T cells and elevated levels of IFN-α are observed in SIV-infected rhesus macaques (which are not natural hosts and develop AIDS) but not in SIV-infected African green monkeys (which are natural hosts and do not progress to AIDS).^33^

Our own studies have demonstrated that ISG expression is not normalized by ART, with elevated levels of ISGs (ISG56, IFI16 and IFI27) observed in CD4^+^ and CD8^+^ T cells isolated from HIV patients on long-term (>34 months) ART when compared with age-matched healthy donors.^34^ The dynamics of ISG expression within different cell subsets is also important as ISG transcript expression was markedly higher in CD4^+^ T cells isolated from patients with low recovery of CD4^+^ T cells. In contrast, ISG transcript expression in CD8^+^ T cells was equally elevated in HIV patients with either low or normal CD4^+^ T-cell counts on ART.^34^

Whether this sustained augmentation of IFN-α activity is beneficial or detrimental to HIV disease progression is a topic of debate and ongoing research study (Table 1). Upon initial infection, IFN-α increases MHC class I and II expression on monocytes, stimulates the development and activation of NK cells and DCs, activates T_H_1 cells and induces differentiation of B cells to plasma cells. Furthermore, IFN-α induces or enhances the expression of restriction factors, which inhibit viral integration and release after reproducing in infected CD4^+^ T cells (see above).

More recently, the role that IFN-α has in establishing chronic immune activation and contributing to the progressive depletion of various lymphocyte populations in the context of HIV disease has been considered. Links between IFN-α activity and T-cell activation are evident in a number of studies. For example, in a cohort of hepatitis C-infected patients treated with pegylated IFN-α over 28 weeks, a progressive upregulation of CD38 expression on CD8^+^ T cells was observed.^26^ Blocking the IFN-α receptors (IFNAR1 and IFNAR2) prevented induction of the activation markers, CD38 and CD69, by HIV-exposed CD4^+^ and CD8^+^ T cells.^35^

In addition, the immunomodulatory functions of IFN-α may also contribute to CD4^+^ T-cell depletion in chronic HIV infection and prevent immune reconstitution. Herbeuval *et al.*^36^ have proposed that IFN-α non-specifically induces the expression of TNF-related apoptosis-inducing ligand (TRAIL) mediated by IFNAR/STAT1 and STAT3 signalling while, simultaneously, the binding of HIV to the CD4 receptor upregulates its ligand, death receptor 5 (DR5). The TRAIL expressed on CD4^+^ T cells and monocytes bind to DR5 and induce apoptosis. This concept is not limited to CD4^+^ T cells as TRAIL-mediated apoptosis has also been implicated in inhibiting memory B-cell reconstitution in successfully treated HIV patients.^37^ *In vitro* exposure of peripheral blood mononuclear cells to IFN-α also upregulates the expression of pro-apoptotic molecules Bak and Fas (CD95) on CD4^+^ T cells and subsequently promotes Fas-mediated apoptosis of T cells from HIV patients.^38^ Bak is upregulated on CD4^+^ T cells in chronic HIV infection, and its expression is associated with CD4^+^ T-cell loss.^38^

Furthermore, our own *in vitro* studies suggest that IFN-α can enhance activation-induced proliferation (via T-cell-receptor stimulation) but inhibit homoeostatic proliferation (IL-7-induced) of T-cells. Both of these effects may adversely affect CD4^+^ T-cell homoeostasis in HIV patients, promoting the loss of T cells by accelerating cell turnover and activation-induced cell death, and decreasing the renewal of T cells by inhibiting the proliferative effects of IL-7.^39^

## Monocyte activation and IFN-α in atherosclerotic vascular disease and neurological disease in HIV patients

Monocytes are innate immune cells of the myeloid lineage that arise from a precursor stem cell population that also produces macrophages and DCs. Within this differentiation pathway, monocytes serve as a circulating source of professional antigen-presenting cells in tissues, including tissue-resident macrophages (such as brain microglial cells) and monocyte-derived DCs. One of the defining properties of monocytes is their ability to respond to infection and inflammation by crossing epithelial barriers to enter tissue compartments, including sites of ‘immune privilege' such as the central nervous system. This is of relevance in the context of HIV infection, as monocytes may promote initial transmission through genital tract epithelial barriers in acute infection,^40^ and are then thought to have a major role in seeding HIV infection in the central nervous system during chronic infection.^41^

Circulating monocytes are generally short-lived with a half-life of around 3 days, although their ‘default' apoptotic programme is readily overcome by inflammatory signals that can then promote differentiation into long-lived macrophages and monocyte-derived DCs, as well as the maturation of longer-lived monocyte populations.^42^ Resolution of inflammation generally leads to resumption of the apoptotic programme in acute infection, although chronic infection (as in HIV infection) and even chronic non-infectious inflammation (as in cardiovascular disease) can lead to persistent skewing of monocyte differentiation from the dominant ‘classical' monocyte population identified by strong expression of CD14 (a lipopolysaccharide receptor) towards ‘non-classical' monocytes that express CD14 and CD16 (a low-affinity Fc receptor) and are capable of increased production of TNF-α and IL-1.^42^ These CD16^+^ monocyte subpopulations are most permissive of HIV infection^43^ and are also capable of transferring infection into the central nervous system.^41^ Interestingly, the phenotype of HIV-induced CD16^+^ monocytes is also reproduced in acute coronary syndromes,^44^ suggesting that chronic HIV infection produces an inflammatory response that may contribute to a range of prevalent long-term diseases, including ‘serious non-AIDS events', where systemic immune activation is a common factor. Analysis of monocyte gene expression in individuals with active HIV-1 infection has demonstrated a dominant IFN-α signature.^32^

It is notable that several monocyte-derived plasma biomarkers have potential clinical relevance in HIV management, including soluble (s) CD14 which is a strong predictor of all-cause mortality in both HIV+ subjects^45^ as well as in the general population.^46^ Importantly, treatment of HIV infection and attainment of undetectable HIV viral load levels does not necessarily correct the abnormal plasma sCD14 signal,^47^ suggesting that there is residual monocyte activation even in the context of what would be considered ‘successful' HIV therapy. Similarly, elevated levels of sCD163 (a monocyte scavenger receptor) have been linked to risk of cardiovascular disease and arterial inflammation^48^ as well as neurocognitive impairment,^49^ including patients on effective ART. Analysis of monocyte gene expression profiles in chronic HIV infection has demonstrated an IFN-α-induced activation phenotype that correlates with markers of brain neuronal injury *in vivo*.^50^

Effective ART can correct some aspects of the chronic inflammatory response in HIV infection, including restoration of plasma IFN-α levels to normal^26^ and reduction in the proportion of CD16-expressing monocytes.^44^ Nevertheless, it is clear that sCD14 and sCD163 levels remain elevated despite HIV treatment and also maintain their associations with adverse cardiovascular and neurological outcomes in this context. Data from the Strategies for Management of Antiretroviral Therapy study demonstrated that subjects with the highest quartile of sCD14 levels had a sixfold higher risk of death than did those in the lowest quartile, with minimal change after adjustment for other inflammatory markers, CD4^+^ T-cell count and plasma HIV RNA level.^45^ One interesting approach to improving these outcomes has been to consider the ability of antiretroviral agents to penetrate monocyte/macrophage cell populations, using a ‘monocyte efficacy score'.^51^ Preliminary findings suggest that this approach provides greater predictive value for cognitive performance during long-term HIV treatment,^51^ so it is hoped that future studies can further validate both determinations of ‘monocyte efficacy' as well as its clinical application.

These data provide a strong argument that innate immune activation, caused in part by increased IFN-α activity, persists throughout the course of HIV infection, albeit with some modification by ART. This aspect of chronic HIV infection, while undoubtedly of prognostic importance,^45^ is currently ‘invisible' in routine clinical management but should not remain so. Understanding the critical (and measurable) components of this response should lead to novel monitoring and therapeutic strategies that have the potential to shift the HIV management paradigm, 20 years after the last revolutionary advance with the introduction of ART.

## Enhancing and controlling IFN-α activity in HIV patients receiving ART: a treatment conundrum in evolution

IFN-α exerts anti-viral activities but also contributes to HIV-induced immune activation. Consequently, both recombinant IFN-α and IFN-α inhibitors are being assessed as therapy to enhance the eradication of HIV infection or immune reconstitution in HIV patients receiving ART.

Administration of pegylated IFN-α-2a therapy for at least 12 weeks, and up to 24 weeks, in patients with HIV infection controlled by ART and a CD4^+^ T-cell count of >450 μl^−1^ resulted in control of HIV replication after ART was ceased in 9 out of 20 (45%) subjects as well as decreased HIV-1 integration.^52^ In another study, HIV-1 DNA levels in CD4^+^ T cells declined in 12 patients with HIV/HCV co-infection after pegylated IFN-α therapy and ribavirin was added to effective ART.^53^ Such findings suggest that IFN-α therapy might be used as a component of eradication therapy in patients who achieve good recovery of CD4^+^ T cells on ART, though clinical trials in larger numbers of patients and for longer periods of time are needed. In contrast, patients with suboptimal CD4^+^ T-cell recovery and/or persistent immune activation may benefit from therapy that inhibits IFN-α activity.

The rationale for examining IFN-α inhibitors in patients with HIV infection is strongly supported by the findings of studies conducted in patients with autoimmune connective tissue diseases. Patients with systemic lupus erythematosus (SLE), rheumatoid arthritis, dermatomyositis, polymyositis and systemic sclerosis exhibit evidence of increased IFN-α activity,^54^ and there is compelling evidence that this has a role in the immunopathogenesis of SLE.^55^ Increased IFN-α activity also appears to be a cause of atherosclerotic vascular disease in patients with SLE.^56, 57^ Inhibition of pDC activation and IFN-α production are dominant effects of the anti-malarial drug hydroxychloroquine,^58^ which has been a fundamental component of therapy for SLE for over 50 years and reduces the risk of atherosclerotic vascular disease.^59^ More recently, interest has focussed on the use of monoclonal antibodies that inhibit the effects of IFN-α. Sifalimumab and Rontalizumab are humanized IgG1κ monoclonal antibodies that neutralize IFN-α and reduce expression of ISGs in tissues and/or blood leucocytes when administered to patients with SLE^60, 61, 62^ or dermatomyositis/polymyositis.^63^

*In vitro* studies have demonstrated that both hydroxychloroquine and chloroquine decrease HIV-induced activation of pDCs through inhibition of Toll-like receptor 7 (TLR-7) signalling, which results in lower expression of the immunosuppressive molecules indoleamine 2,3-dioxygenase and programmed death ligand 1 and reduced IFN-α production.^25, 64^ Hydroxychloroquine may inhibit TLR signalling by binding to nucleic acids and masking their TLR-binding region.^65^ Although inhibition of TLR-7 signalling might have beneficial effects on immune activation, clinical trials in patients with HIV infection suggest that it also has adverse effects on immune responses against viral infections, including HIV infection.

In a nonrandomized clinical trial conducted in 20 HIV patients with persistent CD4^+^ T-cell deficiency despite suppressive ART, administration of hydroxychoroquine was associated with a reduction in several markers of immune activation without increasing HIV replication, even though it had no short-term effect on CD4^+^ T-cell recovery.^66^ Chloroquine also decreased T-cell activation and proliferation in a small randomized controlled trial conducted in 13 HIV patients not receiving ART.^67^ In contrast, a randomized placebo-controlled trial of hydroxychloroquine in 83 untreated HIV patients demonstrated no change in CD8^+^ and CD4^+^ T-cell activation and proliferation, an increase in plasma HIV RNA levels and a decrease in CD4^+^ T-cell counts.^68^ Furthermore, in the latter study, patients receiving hydroxychloroquine experienced more frequent episodes of flu-like illness. Similarly, chloroquine therapy in rhesus macaques with acute SIV infection resulted in a temporary increase in ISGs and poorer recovery of CD4^+^ T cells.^69^ Taken together, the findings of these studies suggest that inhibition of IFN-α production by hydroxychloroquine or chloroquine has detrimental effects on the control of HIV or SIV replication but might suppress immune activation associated with increased IFN-α activity in HIV patients receiving ART that optimally suppresses HIV replication. However, it should be noted that the anti-inflammatory effects of hydroxychloroquine or chloroquine have not been directly related to inhibition of IFN-α. Clinical trials of IFN-α inhibitors, including monoclonal antibodies to IFN-α, are currently being conducted in, or are being planned for, patients receiving ART who have optimally suppressed HIV infection.

## Conclusions

Continuous production of IFN-α is a prominent feature of chronic HIV infection and, like SLE, may contribute to immune activation leading to immune dysfunction and premature bone, brain and atherosclerotic vascular disease. Blocking type I IFN signalling may reduce both the ISG gene signature and disease manifestations. A better understanding of the paradoxical roles of type I IFNs, and the ISGs serving as signposts along the road to disease progression, may provide a more rational therapeutic approach to targeting residual immune activation and dysfunction in HIV patients receiving ART.

## Figures and Tables

**Table 1:**
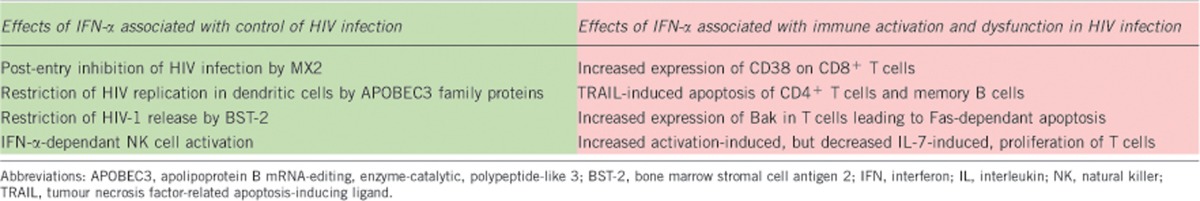
Interferon-**α** exerts both beneficial and deleterious effects on HIV infection
